# Lower urine sodium predicts longer length of stay in acute heart failure patients: Insights from the ROSE AHF trial

**DOI:** 10.1002/clc.23286

**Published:** 2019-11-12

**Authors:** Jonathan W. Cunningham, Jie‐Lena Sun, Finnian R. Mc Causland, Samantha Ly, Kevin J. Anstrom, Joann Lindenfeld, Michael M. Givertz, Lynne W. Stevenson, Neal K. Lakdawala

**Affiliations:** ^1^ Cardiovascular Division, Department of Medicine Brigham and Women's Hospital Boston Massachusetts; ^2^ Duke Clinical Research Institute Duke University Durham North Carolina; ^3^ Renal Division, Department of Medicine Brigham and Women's Hospital Boston MA; ^4^ Vanderbilt University Medical Center Nashville Tennessee

**Keywords:** clinical pharmacology, clinical trials, heart failure, kidney disease

## Abstract

**Background:**

In patients hospitalized with acute heart failure (AHF), low urine sodium concentration (*U*_Na_) after diuretic treatment may identify patients at risk for longer length of stay (LOS) and adverse events. We investigated the prognostic significance of 24‐hour cumulative postdiuretic urine sodium concentration in a multicenter clinical trial population.

**Methods:**

The Renal Optimization Strategies Evaluation AHF (ROSE AHF) trial randomized 360 patients with AHF and renal dysfunction receiving intravenous diuretic to dopamine, nesiritide, or placebo. Sodium concentration was measured in cumulative urine sample collected during the first 24 hours after randomization in 298 patients. Based on prior studies, lower *U*_Na_ was defined as ≤60 mmol/L.

**Results:**

Lower *U*_Na_ was present in 142 (48%) patients, who had longer LOS (7 days vs 5 days, *P* < .001) and less 72‐hour weight loss (5.7 lb vs 9.0 lb, *P* < .001). These associations persisted after controlling for baseline estimated glomerular filtration rate and outpatient furosemide dose. Lower *U*_Na_ did not modify the null effects of dopamine or nesiritide on clinical outcomes. Results were similar for spot rather than cumulative 24‐hour *U*_Na_ concentration.

**Conclusion:**

In patients hospitalized for AHF and renal dysfunction, *U*_Na_ ≤ 60 mmol/L during the first 24 hours of diuresis identifies patients at risk for prolonged hospitalization but does not provide an indication for adjunctive dopamine or nesiritide.

## 1 INTRODUCTION

Intravenous loop diuretic to relieve congestion is the primary therapy for patients hospitalized with acute heart failure.^1^ Persistent congestion, despite treatment with diuretics, is associated with increased risk of adverse outcomes.^2^, ^3^, ^4^, ^5^ Heart failure clinicians need new biomarkers that accurately and efficiently identify patients failing conventional treatment and new therapies to offer such patients.

Urine sodium is a logical candidate biomarker because loop diuretics inhibit sodium reabsorption in the kidney, and thus increase natriuresis. Neurohormonal systems including natriuretic peptides and the renin‐angiotensin‐aldosterone axis closely regulate urine sodium. In patients with normal renal perfusion, urine sodium serves as a barometer for the extracellular volume status; when extracellular volume is low, the kidney responds to conserve sodium. However, in heart failure, renal perfusion is low despite high extracellular volume. Activation of the renin‐angiotensin‐aldosterone axis promotes sodium retention. Thus, lower urine sodium, or persistence of low urine sodium after loop‐diuretic therapy, may reflect more severe heart failure.

Recent single center cohort studies have found that lower urine sodium after loop diuretic administration—either by bolus dosing or continuous infusion—predicts longer length of stay (LOS), worsening renal function, and higher rates of death or rehospitalization for heart failure.^4^, ^6^ Moreover, urine sodium may be a more reproducible measurement of diuretic effectiveness than urine output, which is more commonly used.^7^

The Renal Optimization Strategies Evaluation Acute Heart Failure (ROSE AHF) trial randomized patients admitted for AHF with renal dysfunction to dopamine vs placebo, or nesiritide vs placebo, in addition to loop diuretic therapy.^8^ Renal dysfunction was defined by estimated glomerular filtration rate (eGFR) of 15 to 60 mL/min/1.73 m^2^. The primary trial found no benefit for either adjunctive therapy or that of the pooled placebo group. Urine was collected cumulatively for the first 24 hours after randomization, during which patients received protocol‐driven loop diuretics. In this cohort, total and net sodium excretion values in this period were highly variable, and lower values were associated with higher 6‐month all‐cause mortality.^9^ We hypothesized that patients with lower urine sodium concentration during the first 24 hours after randomization would have longer LOS and might represent a subset of patients who would benefit from dopamine or nesiritide.

## 2 METHODS

The study design and results of the ROSE AHF trial have been published previously.^8^ The study was a double‐blind, placebo‐controlled, randomized trial conducted by the National Heart, Lung, and Blood Institute sponsored Heart Failure Research Network. The study was approved by the institutional review board of each participating center. All participants provided written informed consent.

A total of 360 patients hospitalized with AHF and renal dysfunction at admission, defined as an eGFR of 15 to 60 mL/min/1.73 m^2^ by the Modification of Diet in Renal Disease equation, were enrolled. AHF was diagnosed by at least one symptom (edema, dyspnea, or orthopnea) and at least one sign (edema, rales, ascites, or pulmonary vascular congestion on chest X‐rays). Patients with reduced and preserved ejection fraction were included. Patients were first randomized in a 1:1 ratio to the dopamine or nesiritide strategy, and within each strategy were randomized in a 2:1 ratio to active therapy or placebo for a duration of 72 hours. The dopamine dosage was 2 μg/kg/minute by continuous infusion. The nesiritide dosage was 0.005 μg/kg/minute by continuous infusion. All patients were treated with intravenous loop diuretic at a recommended total daily dose of 2.5 times the total daily outpatient dose based on the results of the DOSE trial,^10^ with a minimum of 80 mg furosemide in the first 24 hours. The total daily dose was administered as two divided bolus doses. Adjustment of the loop diuretic dose and administration of other medications were at the discretion of the treating physicians. Patients with absence of 24‐hour urine sodium data (n = 34) or who received less than the protocol‐specified minimum diuretic dose of 80 mg intravenous furosemide or equivalent in the first 24‐hour period (n = 36) were excluded from this analysis, the latter because urine composition in these patients may not have reflected the effects of diuretic.

Urine was collected in a single container for the first 24 hours after randomization. Sodium concentration was measured from the container of pooled urine. Similar collections were made for the second and third days after randomization. Patients were divided into lower urine sodium (≤60 mmol/L) and higher urine sodium (>60 mmol/L) groups based on prior published data from our institution, which found that patients in the former group had longer LOS, more frequent renal failure, and a greater risk of clinical deterioration.^6^, ^11^ Patients for whom urine sodium measurements were not collected were excluded. Spot urine sodium concentration from a single void was measured 24 hours after randomization; this measurement was used only for secondary analysis.

The primary endpoint of this post hoc analysis was LOS. Secondary endpoints included urine output, weight loss, and serum creatinine change at 72 hours after randomization, congestion score at 7 days after randomization or hospital discharge (whichever occurred earlier), and 60‐day death or rehospitalization for heart failure. Congestion score was defined based on the assessment of orthopnea (≥2 pillows = 2 points; <2 pillows = 0 points) and peripheral edema (trace/mild = 0 points; moderate = 1 point; severe = 2 points).^3^

Baseline patient characteristics and short‐term outcomes are presented by lower and higher urine sodium groups as median with 25th and 75th percentiles for continuous variables and as number with percentage for categorical variables. Categorical variables were compared with chi‐square test or with Fisher's exact test for expected cell counts of 5 or fewer. Continuous variables were compared with Wilcoxon's rank‐sum test. Short‐term outcomes are also presented for each quintile of urine sodium concentration. Linear and Cox regression models measured associations between urine sodium group and clinical outcomes, with or without adjustment for baseline creatinine and outpatient furosemide‐equivalent diuretic dose. Outcomes are also presented for four groups of patients in a 2 × 2 matrix of higher or lower 24‐hour urine output (greater than or less than the median of 2785 mL) and higher or lower urine sodium concentration (greater than or less than/equal to 60 mmol/L); associations between urine sodium and outcomes were measured by linear regression adjusting for urine volume. Interaction tests were performed to assess whether the effects of treatment group on clinical outcomes were modified by the 24‐hour urinary sodium. The linearity and proportional assumptions were satisfied for all the variables in the models. Two‐tailed *P* values of <.05 were considered significant. All statistical analyses were performed using SAS version 9.4 (SAS Institute, Cary, North Carolina). Clinical Trial Registration is available at https://clinicaltrials.gov/ct2/show/NCT01132846 with Unique Identifier No. NCT01132846.

## 3 RESULTS

Of the 360 patients randomized in the ROSE AHF trial, 298 met criteria for this analysis. The mean urine sodium concentration was 62.9 mmol/L (SD 25.9 mmol/L); the range was 20 to 135 mmol/L. Urine sodium concentration for cumulative urine from the first 24 hours was ≤60 mmol/L in 142 patients (48%) and >60 mmol/L in 156 patients (52%). Baseline characteristics of the higher and lower urine sodium concentration groups are shown in Table 1. Patients with lower urine sodium were younger and had lower systolic blood pressure, higher outpatient furosemide‐equivalent dose, slightly lower serum sodium, and higher blood urea nitrogen. The higher and lower urine sodium groups did not differ significantly with respect to the cause of HF, left ventricular ejection fraction, New York Heart Association (NYHA) functional class, the use of neurohormonal antagonists, the degree of congestion on admission, or admission of NT‐pro‐BNP or serum creatinine.

**Table 1:** Baseline characteristics of the lower and higher urine sodium groups

Characteristic	Urinary sodium ≤ 60 mmol/L (N = 142)	Urinary sodium > 60 mmol/L (N = 156)	*P* value
Age (years)	68 (59‐79)	72 (63‐80)	.034
Male gender	104 (73%)	121 (78%)	.386
Race			.755
Black	29 (20%)	28 (18%)	
White	106 (75%)	122 (78%)	
Other	7 (5%)	6 (4%)	
Weight (lb)	200 (170‐247)	199 (171‐238)	.415
Body mass index	31.4 (27.1‐37.3)	30.0 (26.5‐35.4)	.185
Ejection fraction (n = 297)	35 (20‐54)	33 (23‐52)	.930
Ejection fraction ≤40% (n = 297)	88 (62%)	94 (61%)	.815
Orthopnea (n = 286)	122 (90%)	133 / (88%)	.534
Systolic blood pressure (mmHg)	110 (100‐122)	117 (109‐131)	<.001
Heart rate (beats/min)	74 (67‐84)	74 (65‐83)	.633
JVP (n = 285)			.873
<8 cm	5 (4%)	8 (6%)	
8‐12 cm	27 (19%)	26 (18%)	
13‐16 cm	50 (36%)	54 (37%)	
>16 cm	57 (41%)	58 (40%)	
Comorbidities			
HF hospitalization in last yr (n = 296)	95 (67%)	109 (71%)	.471
Ischemia as cause of HF	80 (56%)	91 (58%)	.728
Atrial fibrillation/flutter	87 (61%)	95 / (61%)	.948
Diabetes	87 (61%)	81 / (52%)	.104
ICD	60 (42%)	67 / (43%)	.904
COPD	39 (28%)	40 / (26%)	.722
NYHA functional class (n = 287)			.178
II	3 (2%)	9 (6%)	
III	97 (71%)	96 (64%)	
IV	36 (27%)	46 (31%)	
Medications at enrollment			
ACE inhibitor or ARB	60 (42%)	83 (53%)	.059
Beta blockers	117 (82%)	130 (83%)	.830
Aldosterone antagonist	46 (32%)	43 (28%)	.363
Outpatient furosemide‐equivalent dose, mg/day (n = 285)	120 (80‐160)	80 (40‐120)	<.001
Laboratory values at baseline			
Serum sodium (mg/L)	138 (135‐141)	139 (137‐141)	.002
NT‐pro BNP, pg/mL (n = 291)	4290 (1760‐9511)	6149 (3120‐10 422)	.072
Blood urea nitrogen, mg/dL (n = 296)	42.0 (28.0‐57.0)	34.0 (26.6‐47.6)	.021
Creatinine, mg/dL (n = 291)	1.70 (1.38‐2.03)	1.59 (1.30‐1.98)	.107
eGFR (mL/min/1.73 m^2^)	40.4 (30.8‐51.5)	43.9 (32.2‐55.7)	.128
Cystatin C, mg/L (n = 291)	1.7 (1.5‐2.3)	1.7 (1.4‐2.2)	.278
Bicarbonate, mEq/L (n = 276)	28.0 (24.2‐30.3)	27.0 (24.0‐30.0)	.470

*Notes*: Categorical variables expressed as n (%). Continuous variables expressed as median (interquartile range). For variables with incomplete data, the number of patients with available data is indicated; percentages for categorical variables reflect the number of patients with available data in the denominator.

Abbreviations: ACE, angiotensin‐converting enzyme; ARB, aldosterone receptor blocker; CAD, coronary artery disease; CKD, chronic kidney disease; Cr, creatinine; Hct, hematocrit; ICD, implantable cardiac defibrillator; NT‐pro BNP, n‐terminal prohormone of brain natriuretic peptide; eGFR, estimated glomerular filtration rate.

Short‐term outcomes for patients with lower or higher urine sodium concentration are presented in Table 2. In the lower urine sodium group, LOS was longer (median 7 vs 5 days, *P* < .001) and more patients had persistent congestion at day 7 or discharge (49% vs 37%, *P* = .03). Patients with lower urine sodium lost less weight at 72 hours (median 5.7 lb vs 9.0 lb, *P* < .001), although there was no statistically significant difference in urine output (7.8 L vs 8.2 L, *P* = .20). Urine sodium remained a significant predictor of LOS and weight loss after controlling for baseline eGFR and outpatient furosemide‐equivalent dose in a multivariable model. These relationships were significant when urine sodium was expressed as a continuous variable: adjusted LOS was 0.5 days longer and adjusted 72 hours weight loss was 0.5 lb lower per 10 mmol/L decrease in urine sodium (*P* < .001 for both). The associations were also observed when urine sodium concentration was measured from spot rather than cumulative sample at 24 hours: adjusted LOS 2.4 days was longer (*P* = .001) and adjusted weight loss 3.2 lb was lower (*P* < .001) in the lower urine sodium group.

**Table 2:** Short‐term outcomes by urine sodium group

Characteristic	Urinary sodium ≤60 mmol/L (N = 142)	Urinary sodium > 60 mmol/L (N = 156)	*P* value
Length of stay, d (n = 293)	7.0 (5.0,12.0)	5.0 (4.0,7.0)	<.001
Length of stay > 7 d (n = 293)	62 (45%)	35 (23%)	<.001
Persistent congestion at discharge^a^ (n = 289)	67 (49%)	56 (37%)	.030
72 h urine volume, mL (n = 265)	7800 (5625,10 140)	8212 (6638,10 200)	.196
72 h weight loss, lb (n = 284)	5.7 (2.2,10.6)	9.0 (4.8,13.2)	<.001
Cre increase > 0.3 mg/dL at 72 h (n = 269)	18 (14%)	31 (23%)	.064

Abbreviation: Cre: creatinine.

a If congestion score > 0 at discharge or 7 days after randomization. Categorical variables expressed as n (%). Continuous variables expressed as median (interquartile range). For variables with incomplete data, the number of patients with available data is indicated; percentages for categorical variables reflect the number of patients with available data in the denominator.

**Figure 1:**
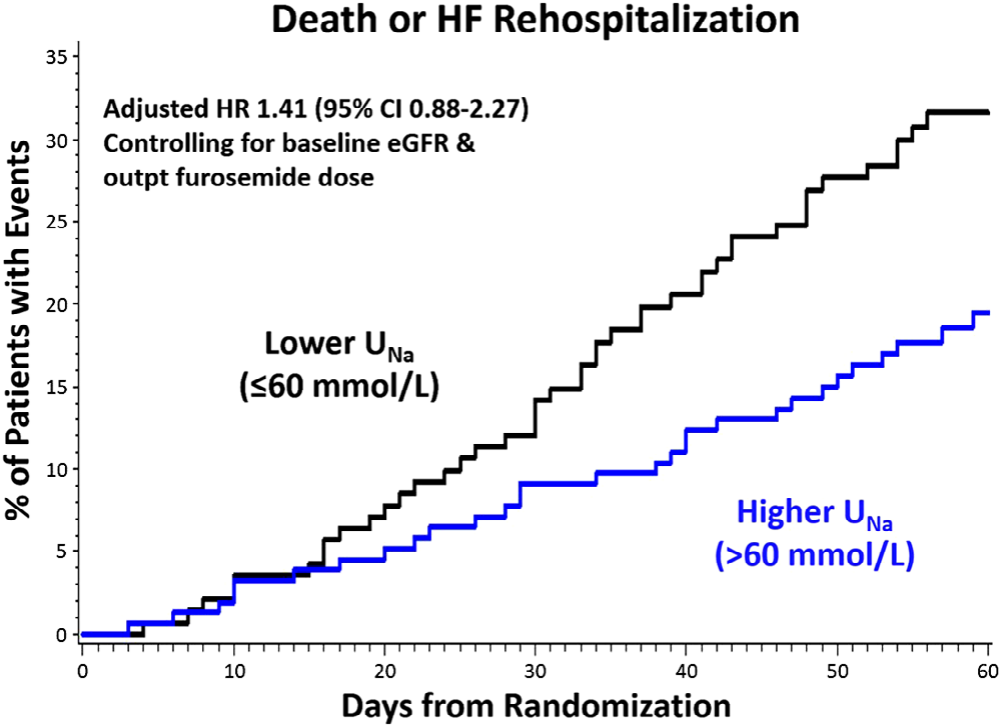
Time to death or heart failure rehospitalization in higher and lower urine sodium groups. eGFR: estimated glomerular filtration rate; HR, hazard ratio; *U*_Na_: urine sodium concentration

A trend toward a higher rate of death or heart failure rehospitalization in the lower urine sodium group did not reach statistical significance. This composite outcome occurred in 45 patients (32%) in the lower urine sodium group and 31 patients (20%) of patients in the higher urine sodium group at 60 days. In a Cox regression model adjusting for baseline eGFR and outpatient furosemide‐equivalent dose, the hazard ratio for lower urine sodium was 1.41 (95% CI 0.88‐2.27) for death or heart failure rehospitalization (Figure 1).

**Figure 2:**
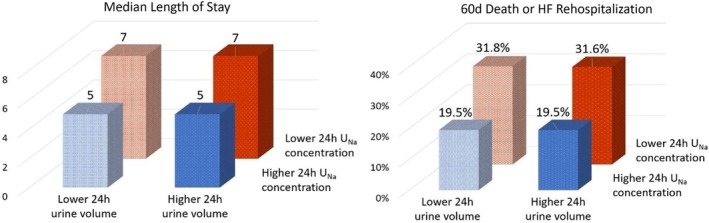
Prognostic significance of urine sodium concentration and urine volume at 24 hours. Lower urine sodium concentration defined as ≤60 mmol/L. Lower urine volume defined ≤2875 mL, which was the median value. Rate of 60 days death or HF rehospitalization refers to Kaplan‐Meier event rate. HF, hazard ratio; LOS, length of stay

Urine sodium concentration at 24 hours was a more powerful predictor of LOS than urine volume at 24 hours. Patients in the lower urine sodium group had a longer LOS (7d vs 5d) regardless of whether 24‐hour urine output was above or below the median value (Figure 2). After controlling for dichotomized 24‐hour urine sodium concentration, 24‐hour urine volume was not associated with LOS (*P* < .001 for urine sodium concentration, and *P* = .69 for urine volume in a linear regression model including both variables).

**Figure 3:**
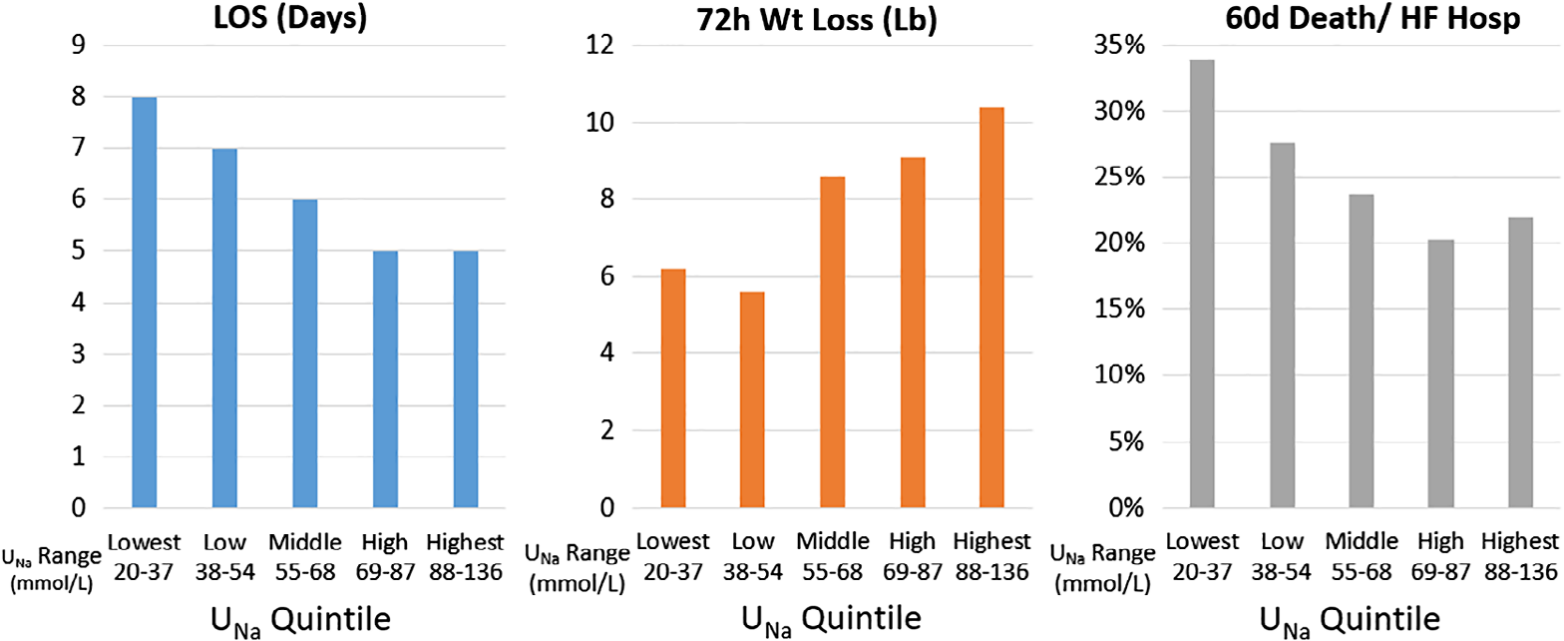
Outcomes by quintile of 24‐h urine sodium concentration. Median values presented for LOS and 72‐hour weight loss. The range of urine sodium concentration for patients included in each quintile is provided below the corresponding bar. LOS, length of stay; *U*_Na_, urine sodium concentration

Outcomes across 5 quintiles of urine sodium concentration are shown in Figure 3. The relationships between lower urine sodium and longer LOS, greater weight loss, and higher rates of death or HF rehospitalization were consistent across the range of urine sodium.

Urine sodium concentration at 24 hours did not modify the null effect of nesiritide and dopamine on clinical outcomes (*P* interaction of >.05 for all outcomes). Among patients with urine sodium ≤60 mmol/L, there was no significant difference between dopamine and placebo or nesiritide and placebo with respect to LOS, urine output, weight loss, change in creatinine, or 60‐day survival free of rehospitalization (Table S1). There was a trend toward lower 60‐day survival free of rehospitalization in patients with lower urine sodium randomized to nesiritide compared with placebo that did not reach statistical significance (*P* interaction of .06 unadjusted, .053 adjusted for eGFR, and furosemide‐equivalent dose). As previously reported, among the patients with left ventricular ejection fraction ≤40%, patients randomized to dopamine had lower rates of death or heart failure rehospitalization compared with placebo.^12^ This relationship was not modified by urine sodium concentration.

## 4 DISCUSSION

The major finding of this study is that lower urine sodium concentration predicted less weight loss and longer LOS in a cohort of patients hospitalized with AHF and renal dysfunction who were managed with protocol‐driven loop diuretic. The relationship between urine sodium and outcomes was consistent across the range of urine sodium values, without a clear threshold. Urine sodium concentration was more predictive of LOS than urine volume at 24 hours. These results suggest that lower urine sodium identifies diuretic‐refractory patients who may require escalation of therapy. However, patients with lower urine sodium did not benefit from adjunctive dopamine or nesiritide.

This study is consistent with and extends prior reports demonstrating that urine sodium is a marker of diuretic effectiveness. Testani and colleagues found that a prediction model including urine sodium concentration for 2 hours after diuretic administration, eGFR, and serum/urine creatinine ratio accurately predicted cumulative 6‐hour sodium excretion and urine output.^7^ Single‐center cohort studies have demonstrated that lower urine sodium after loop diuretic predicts worsening heart failure, defined in various reports as worsening renal function, delayed symptom improvement, or need for inotrope or mechanical support.^4^, ^6^, ^13^ Our study adds to this literature in several ways. First, sample size was significantly larger: nearly 300 patients compared with 50 to 100 patients in single‐center studies. Second, loop diuretic was administered according to a standardized protocol—2.5 times the patient's home furosemide‐equivalent daily dose, divided between two bolus doses—eliminating variation in dose due to patient or provider factors. The prognostic significance of urine sodium concentration was independent of the baseline furosemide‐equivalent diuretic dose. Third, clinical outcomes were adjudicated according to prespecified criteria in the context of a clinical trial.

Our findings complement a recent report by Hodson et al from the same cohort, which found that total urinary sodium excretion and net sodium excretion are associated with 6‐month all‐cause mortality.^9^ This study adds short‐term outcomes such as weight loss and LOS, which underscore that urinary sodium is a marker of the effectiveness of current diuretic dose in addition to long‐term heart failure prognosis. We also demonstrate that both cumulative and spot urine sodium concentration at 24 hours are associated with LOS and weight loss. Spot and total urine sodium concentration may be complementary tests: spot urine sodium concentration yields actionable information more quickly, while cumulative urine sodium concentration integrates the rate of natriuresis during periods between diuretic doses. Notably, spot urine in this study remained useful despite collection at 24 hours rather than at a precisely specified interval after diuretic administration.

We chose to focus on spot and cumulative urine sodium concentration rather than total urinary sodium content because urine output is difficult to measure and was poorly correlated with outcomes in our study. We found that 24‐hour urine volume was not associated with LOS after adjustment for 24‐hour urine sodium concentration. Others have reported that urine output recorded by bedside nurses is often inconsistent with gold standard measurements by research coordinators and with changes in weight.^7^, ^14^ The robust association of cumulative urine sodium concentration with short‐term outcomes in our study may be due to lower sensitivity to missed voids.

A second finding of this study is that lower postdiuretic urine sodium did not identify a population of AHF patients who benefitted from addition of dopamine or nesiritide compared with placebo. Some have attributed the neutral result of the ROSE AHF trial to enrollment of subjects who were not truly refractory to diuretics, as reflected by the average 72‐hour urine output of 8.3 L.^7^ Our results do not support this hypothesis. Unfortunately, other potential therapies for diuretic‐resistant patients such as ultrafiltration, tolvaptan, or spironolactone have not shown benefit.^15^, ^16^, ^17^ Addition of a thiazide diuretic may be an effective strategy for these patients because increased sodium reabsorption in the distal convoluted tubule is a frequent cause of diuretic resistance.^18^, ^19^ Development of a better medical therapy for diuretic‐resistant patients is an important research priority.

Our study should be interpreted in light of two limitations. First, the timing of urine collection after randomization could have introduced bias into the comparison of the treatments among the lower urine sodium subgroup. Dopamine or nesiritide might increase urine sodium concentration, thereby reassigning patients to the lower risk group and biasing assessment of drug benefit toward the null. However, the data do not support this effect; in fact, median urine sodium concentration was the highest in the placebo group. Patients receiving dopamine (n = 51) and nesiritide (n = 48) were overrepresented in the lower urine sodium group compared with patients receiving placebo (n = 43). Second, other factors not measured or controlled in this study—non‐osmotic vasopressin release, acute kidney injury, glycosuria, and variations in dietary sodium intake—also influence urine sodium. Metabolic alkalosis, which may occur in this population due to diuretic‐associated volume contraction, may also confound interpretation of urine sodium.

In conclusion, lower urine sodium concentration during the first 24 hours of protocol‐driven intravenous loop diuretic therapy was associated with less weight loss and longer LOS among patients with acute heart failure and renal dysfunction. Patients with lower urine sodium did not benefit from adjunctive therapy with dopamine or nesiritide. Urine sodium is a promising biomarker, which may assist in the early identification of diuretic‐resistant patients. Further research is needed to identify effective adjunctive therapies for this population.

## CONFLICT OF INTERESTS

Dr Lindenfeld has served as a consultant to Boehringer‐Ingelheim, Novartis, Relypsa, Abbott Vascular, Edwards Lifesciences, VWave, CVRx, and Impulse Dynamics, and received a grant from Astra Zeneca. Dr. Mc Causland is supported by National Institute of Diabetes and Digestive and Kidney Diseases grant K23DK102511. The other authors report no conflicts.

## Supporting information


**Table S1**Short‐term outcomes in patients with urine sodium <=60 randomized to dopamine, nesiritide, or placebo
