# Supplementary material for: Lower urine sodium predicts longer length of stay in acute heart failure patients: Insights from the ROSE AHF trial
Source: Clin Cardiol. 2019 Nov 12;43(1):43–9. doi: 10.1002/clc.23286 (PMC6954375; doi:10.1002/clc.23286)
Supplement: Supplementary file 1 — Table S1Short‐term outcomes in patients with urine sodium <=60 randomized to dopamine, nesiritide, or placebo [file CLC-43-43-s001.docx]

**Supplemental Table: Short-term outcomes in patients with urine sodium <=60 randomized to dopamine, nesiritide, or placebo**

|  | **Placebo (n=43)** | **Dopamine (n=51), p vs placebo** | | **Nesiritide (n=48), p vs placebo** | |
| --- | --- | --- | --- | --- | --- |
| Length of stay, d (n=139) | 7.5 (6.0,14.0) | 7.0 (5.0,11.0) | 0.30 | 7.0 (5.0,11.5) | 0.19 |
| Length of stay >7d (n=139) | 21 (50%) | 21 (43%) | 0.50 | 20 (42%) | 0.43 |
| Persistent congestion at discharge* (n=136) | 20 (49%) | 26 (53%) | 0.69 | 21 (46%) | 0.77 |
| 72h urine volume, mL (n=137) | 7225 (5700,10300) | 7600 (5475,9538) | 1.0 | 8700 (6300,10775) | 0.39 |
| 72h weight loss, lbs (n=139) | 4.4 (1.4,9.3) | 5.7 (2.0,10.4) | 0.69 | 5.9 (3.5,10.6) | 0.26 |
| Cre rise >0.3 mg/dL at 72h (n=131) | 2 (5%) | 8 (17%) | 0.10 | 8 (17%) | 0.17 |

*If congestion score>0 at discharge or 7d after randomization. Categorical variables expressed as n (%). Continuous variables expressed as median (interquartile range). For variables with incomplete data, the number of patients with available data is indicated; percentages for categorical variables reflect the number of patients with available data in the denominator.

Cre: creatinine;
